# Evaluating the role of active TGF-*β*1 as inflammatory biomarker in Kashmiri (North-Indian) patients with systemic sclerosis: a case-control study

**DOI:** 10.1186/s42358-024-00433-4

**Published:** 2024-12-23

**Authors:** Sakeena Ayub, Tabasum Shafi, Roohi Rasool, Mushtaq A. Dangroo, Muzaffar A. Bindroo, Ayaz Gull, Lamya Ahmad Al-Keridis, Nawaf Alshammari, Mohd Saeed, Zafar Amin Shah

**Affiliations:** 1 Department of Immunology & Molecular Medicine, Sher-I-Kashmir Institute of Medical Sciences (SKIMS), Soura, Srinagar, Jammu and Kashmir 190011 India; 2 Division of Rheumatology, Department of Internal Medicine, Sher-I-Kashmir Institute of Medical Sciences (SKIMS), Soura, Srinagar, 190011 India; 3 Department of Biology, Faculty of Science, Princess Nourah Bint Abdulrahman University, Riyadh, 11671 Saudi Arabia; 4 Department of Biology, College of Science, University of Hail, Hail, Saudi Arabia

**Keywords:** Systemic sclerosis, TGF-*β*1, mRNA expression, ELISA, qRT-PCR

## Abstract

**Background:**

As a master immune system regulator, transforming growth factor *β*1 (TGF-*β*1) is closely linked to the complicated pathophysiology and development of systemic sclerosis (SSc), a multisystem fibrotic disease.

**Objective:**

We aim to evaluate the transcriptional levels of TGF-*β*1 mRNA in PBMCs, assess the TGF-*β*1 serum levels of SSc patients, and compare them with those of healthy subjects.

**Methods:**

PBMCs were isolated from whole blood of 50 SSc patients and in 30 healthy controls. After total RNA was extracted from isolated PBMCs, complementary DNA (cDNA) synthesis was performed. Afterward, the expression of TGF-*β*1 mRNA was assessed using quantitative real-time PCR using the SYBR Green, GAPDH, and TGF-*β*1 specific primers. The serum levels of TGF-*β*1 were determined using a commercially available ELISA kit.

**Results:**

There was a significant upregulation of TGF-*β*1 relative expression (*p* < 0.0001), when SSc patients were compared to the control group. The diffuse subgroup was more common in patients with elevated TGF-*β*1 mRNA expression (*p* < 0.0001). However, an insignificant difference was observed between the disease subsets of SSc. Serum TGF- *β*1 levels were upregulated in SSc patients (78.35 ± 23.16) compared to healthy subjects (61.06 ± 15.90), and were considerably higher in SSc patients with ILD (*p* < 0.01) and positive anti-topo-Isomerase antibody (*p* < 0.0001).

**Conclusion:**

In patients with SSc, elevated levels of TGF-*β*1 in serum and their correlation with clinical symptoms imply that this cytokine may serve as a marker for fibrotic and vascular involvement in SSc.

## Introduction

Systemic Sclerosis (SSc) is a rare heterogeneous connective tissue disorder that causes fibrosis and abnormalities in the immune system and circulatory system, which can lead to various systemic and dermatological problems [1]. The heterogeneity and wide variation in organ involvement, rate of disease progression and survival between patients are prominent characteristics of the disease. SSc is further classified into limited and diffuse cutaneous SSc based on EULAR/ACR classification criteria [2]. In the case of a limited cutaneous subset (lcSSc), skin thickening is restricted to the distal extremities, with minimal systemic involvement, and in cases of the diffuse cutaneous subset (dcSSc), skin thickening is extensive, and systemic involvement is significant [3]. Effective management of systemic sclerosis requires early diagnosis and ongoing disease progression monitoring. Although a definitive cure does not exist for systemic sclerosis, treatment primarily focuses on managing affected organs and alleviating symptoms to prevent further organ damage in individuals with systemic sclerosis. Although the disease process may affect any organ, one of the main causes of morbidity and death in SSc is still the pulmonary consequences, such as pulmonary hypertension (PH) and interstitial lung disease (ILD) [4–6]. In fact, combined, ILD and PH account for 60% of mortality associated with SSc [7]. Treatment is primarily focused on an intensive immunosuppressive medication that is specifically recommended in the progressive types of SSc-ILD [8–10], in contrast to what is observed in IPF. Finding patients who are more likely to develop ILD and need early intervention is one of the biggest issues facing clinicians [11–13]. Prognostic biomarkers are essential in this case to assist clinicians in predicting the onset of ILD and administering appropriate treatment.

Since identifying TGF-*β*1 a strong profibrotic and immunomodulatory properties, it has been acknowledged as a major player in the pathophysiology of the fibrotic process in SSc and other fibroproliferative diseases [3, 14]. The transforming growth factor- *β* (TGF-*β*) is a class of pleiotropic cytokines often encoded in three different mammalian isoforms: TGF-*β*1, TGF-*β*2, and TGF-*β*3 [15]. Out of the three isoforms, the TGF-*β*1 homodimer is the most widely studied subtype and has diverse outcomes on a wide range of immune and non-immune cell types. One of most important actions of TGF-*β*1 is to stimulate the synthesis and development of several extracellular matrix components linked to tissue fibrosis. TGF-*β*1 also promotes the synthesis of protease inhibitors such as tissue inhibitors of metalloproteinase-1 but suppresses the formation of metalloproteinases, which hydrolyze collagen [16]. TGF-*β*1 causes tissue-resident fibroblasts to differentiate into myofibroblast-activated cells, which can produce and express large amounts of muscle actin and have a variety of differentiated phenotypes. TGF-*β*1 is an essential factor in tissue fibrosis, and measurements of its serum levels can be used to determine how active the fibrotic process is [17]. Although research on TGF-*β* inhibition as a therapy option for SSc is ongoing, the findings have been inconsistent [14]. Given TGF-*β*1’s significant involvement in tissue fibrosis, measures of its blood levels may indicate how active the fibrotic process is. Thus, the current study aimed to investigate the TGF-*β*1 mRNA expression in the serum and peripheral blood mononuclear cells (PBMC) of individuals with systemic sclerosis compared to that of healthy controls, and to correlate the levels to clinical features mainly with ILD and PH.

## Methods

### Study subjects

The current study was conducted in Immunology and Molecular Medicine in collaboration with the Department of Internal Medicine, Division of Rheumatology, Sher-I-Kashmir Institute of Medical Sciences (SKIMS), Soura, Srinagar (J&K), a tertiary care referral hospital in North India. Proformas were used to document clinical characteristics at the time of evaluation.

#### Inclusion and exclusion criteria

A total of 50 naïve SSc cases were recruited into the study. At the entry, patients were profiled into dcSSc and lcSSc subgroups linked to distinct clinical consequences and prognoses were made at the point of admission using the criteria proposed by Van den Hoogen et al. 2013 and updated by the European League Against Rheumatism [2]. All the patients followed American Rheumatological Criteria in our study, and hospital investigations were used to determine the clinical profile of these individuals. Following internationally agreed guidelines, SSc was defined. SSc with skin injury can be classified as either limited cutaneous SSc (lcSSc), in which the injury does not go beyond the elbows and knees, or diffuse cutaneous SSc (dSSc) in which skin injury affects the proximal limbs, trunk, and/or both. Standard assessment exams include respectively: Rodnan score, cardiac ultrasound, lung CT scan, PFT, and capillaroscopy. Sc-ILD was defined by a combination of specific HRCT images of at least 10% of all parenchyma (reticulations, honeycombing and/or ground glass opacities) with clinical signs or symptoms (cough, shortness of breath) and alteration of PFTs. Following multiple discussions with clinicians, all cases were validated to determine whether or not SSc-ILD was present. Echocardiography, the forced vital capacity (FVC)/ diffusion capacity for carbon monoxide ratio (DLCO) were used to achieve early diagnosis of SSc-PAH. All of the patients’ clinical, laboratory, and demographic information was recorded using our standard protocol. The anti-topoisomerase I (ATA), anti-centromere antibody (ACA), and anti-nuclear antibody (ANA) data of an autoantibody profile in SSc patients were obtained from the test results of the patients.

The study excluded immunosuppressed patients, those with overlapping syndromes (SSc with Systemic lupus erythematosus or SSc with Rheumatoid arthritis), and also pregnant or nursing women.

#### Control subjects

Thirty healthy Kashmiri volunteers who were matched for age, geographical region, gender, and ethnicity and had no history of autoimmune, inflammatory, or other disorders were included in the study.

### Isolation of primary human peripheral blood mononuclear cells

After the diagnosis, peripheral blood was extracted by venipuncture, collected into ethylenediaminetetraacetic acid (EDTA) tubes, centrifuged for 10 min at 1500× *g* at 4 °C by using density centrifugation using Ficoll (Ficoll Isopaque, HIMEDIA).

#### RNA isolation and real-time polymerase chain reaction (qRT-PCR)

**Fig. 1:**
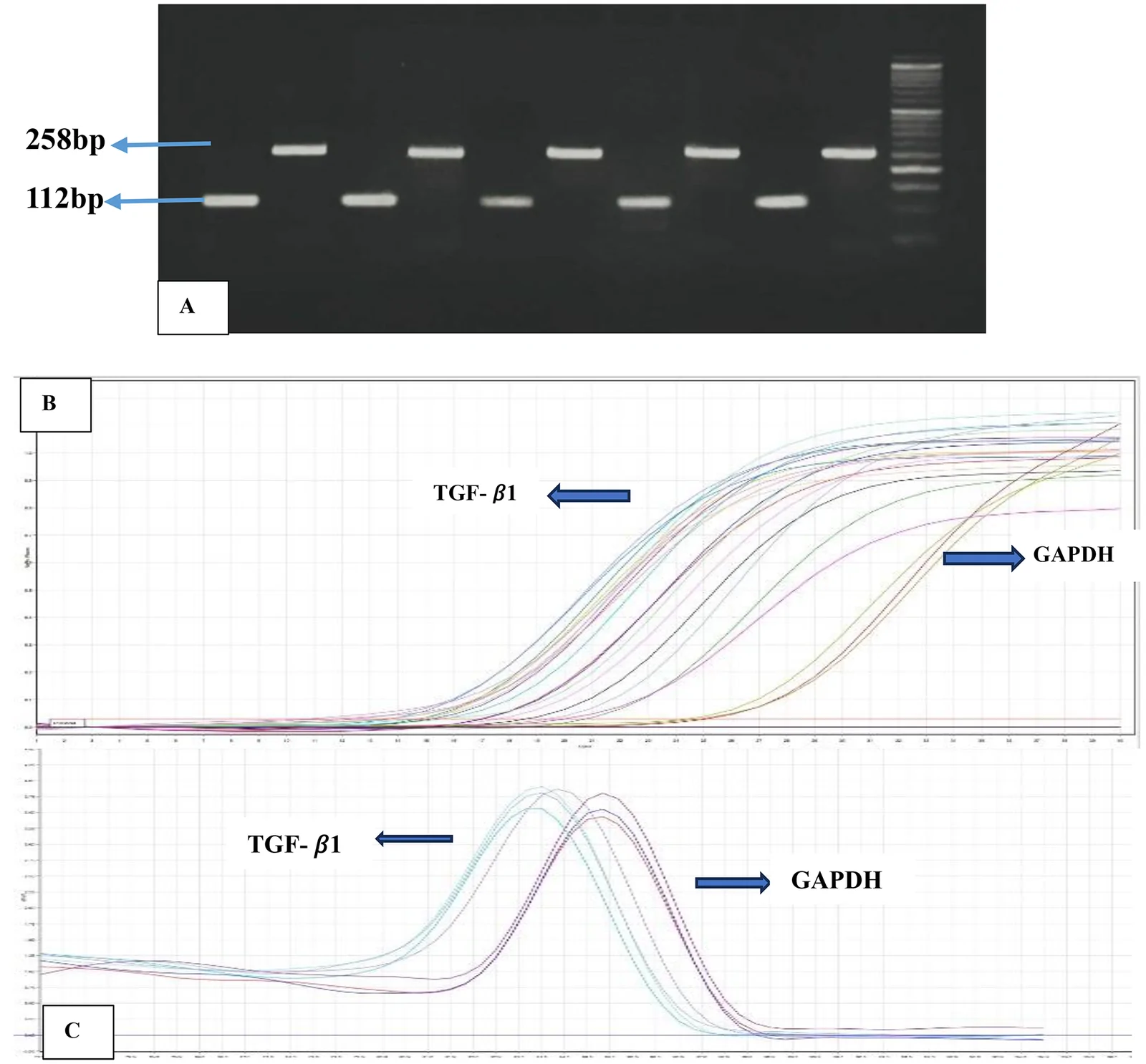
Representative pictures of TGF-β1 qPCR analysis in 50 SSc cases: **A**-qPCR post amplification gel picture of TGF-β1 (112bp) and GAPDH (258bp), lane M: 100bp marker. **B**-Amplification plot of TGF-β1 and GAPDH. **C**-Melting curve analysis of TGF-β1 and GAPDH

In both cases and controls, total RNA was isolated from Primary Human PBMCs using TRIzol (Invitrogen), as per the manufacturer’s instructions. After extraction, DNA contamination was removed by using DNase treatment (ThermoScientific). Relative absorbance values (A260/280) were used to evaluate the extracted RNA’s purity, and the ratio was found to be ~ 2. The extracted RNA’s integrity was checked on a 1% Agarose gel prepared in DEPC-treated water. 200 ng/sample of total RNA was reverse transcribed, by using the Maxima First strand cDNA synthesis kit (ThermoScientific K1641) as directed by the manufacturer. After that, by using gene-specific primers, 2 ul of cDNA as a template, and Maxima SYBR Green/ROX qPCR Master Mix (Thermo Scientific K0221), TGF-*β*1 mRNA levels were determined by quantitative real-time Polymerase Chain Reaction (qRT-PCR) (Rotor-Gene Qiagen thermocycler). Primer concentration and annealing temperature were first standardized by a routine gradient PCR utilizing cDNA as a template. The absence of non-specific amplification products and primer dimers was ensured for proper quantification. To determine the relative expression of each target for the qPCR data analysis, the internal *GAPDH* expression level was normalized using the 2^−ΔΔCt^ method [18]. *GAPDH* was selected as the reference gene as it showed a lesser deviation of Ct values as compared to the other two housekeeping genes- *β-actin* and *HPRT* that were tested in our study. The successful RT-PCR amplification was assessed by resolving the amplicon on 3% agarose gel shown in Fig. 1A and the amplification plot is shown in Fig. 1B). A post-amplification melting-curve analysis was performed (Fig. 1C) to ensure reaction specificity and assess real-time PCR reactions for primer-dimer artifacts. Representative pictures of TGF- *β*1 qPCR analysis are shown in Fig. 1.

### Assessment of serum TGF-*β*1 levels

Blood samples were centrifuged and serum was separated from the samples. Before usage, all serum samples were kept at -70∘C. Using a particular ELISA kit, the amounts of active TGF-*β*1 in serum and culture supernatants were measured per the manufacturer’s instructions (DRG, UK). As directed by the manufacturer, serum samples were acidified with HCl for one hour while being run in duplicate. This enables the release of the naturally active form of TGF-β1 from the biologically inactive complex that is created when a mature TGF-β dimer and a second dimer (latency-associated protein) bind non-covalently.

### Statistical Analysis

Prism 8 software (Graphpad Inc., San Diego, USA) and SPSS version 21.0^®^ (IBM, Chicago, USA) were used for data analysis. The parametric tests (student t-test and one-way ANOVA were used to assess the statistically significant difference among two and multiple groups, respectively), and the Shapiro-Wilks test was used to confirm the normality of the samples. Serum cytokine levels were compared using the Mann-Whitney U test. The correlation between the variables was assessed using the Pearson or Spearman correlation test. A statistical significance threshold of 5% (*p* < 0.05) was used.

## Results

### Basic characteristics of study subjects

The current study included a total of 80 subjects (50 patients with scleroderma and 30 normal controls) for TGF-*β*1 expression analysis. Out of these, there were 90% female patients and 10% male patients. Most of the patients were found in rural areas (62.5%). On average, the patients were 37.11 ± 9.61 years old. The Raynaud’s Phenomenon average onset time was 4.87 ± 2.62-years. The mean duration of the disease was 5.3 ± 3.3 years. When patients were stratified into subtypes, the diffuse type of the disease was diagnosed in 38 (76%) patients whereas the limited form was in 12 (24%) patients. The mean age of patients in the limited subtype of SSc was 36.81 ± 10.91 years, whereas the mean age of patients in the diffused subtype of SSc was 36.02 ± 8.20 years. Indicating the proper frequency matching, the mean age and gender distributions of cases and controls did not differ substantially. Their basic characteristics are outlined in Table 1. Concomitant disorders that were found in the patients were oesophageal dysmotility, digital ulcers, arthralgia, arterial hypertension, and pulmonary arterial hypertension.

**Table 1:** Basic characteristics of SSc cases and their antibody profile

Characteristics	Number
Age (yrs); mean ± SD	37.11 ± 9.61
**Dwelling (N = 50)**	
Rural	35 (70%)
Urban	15 (30%)
**Gender (N = 50)**	
Female	48 (96%)
Male	02 (04%)
Disease Duration in Years	5.3 ± 3.3
**Clinical subgroups**	
LcSSc	12 (24%)
dcSSc	38 (76%)
**Autoantibodies (N = 50)**	
ANA	40 (80%)
ACA	12 (24%)
ATA	38 (76%)
Lung fibrosis	30 (60%)
PAH	28 (56%)
Digital ulcers	28 (56%)
Sclerodactyly	45 (90%)
Puffy fingers	31 (62%)
Gastrointestinal manifestations	25 (50%)

lcSSc: limited cutaneous SSc, dcSSc: diffuse cutaneous SSc, PAH; pulmonary arterial hypertension; ILD: interstitial lung disease, ATA: anti-topoisomerase I, ACA: anti-centromere antibody, ANA: anti-nuclear antibody

### Autoantibodies and disease manifestations in SSc

The clinical appearance of the assessed SSc patients showed that 95% had skin thickening. 92% of patients experienced arthralgia, telangiectasia, and sclerodactyly. Approximately 95% of patients had Raynaud’s phenomenon observed at the initial assessment before the commencement of the disease. The main clinical features of SSc patients are listed in Table 1. In our study, we found that 60% of patients had interstitial lung disease or pulmonary fibrosis, while 56% of patients had pulmonary arterial hypertension. 80% of SSc patients had positive ANA tests, 24% had ACA positivity, and 76% of patients were found to be ATA positive (Table 1). Calcinosis, arthralgia, and telangiectasia were more common in the ACA group of patients, while pulmonary fibrosis was more common in the ATA group, though the difference was not statistically significant.

### mRNA expression analysis

**Fig. 2:**
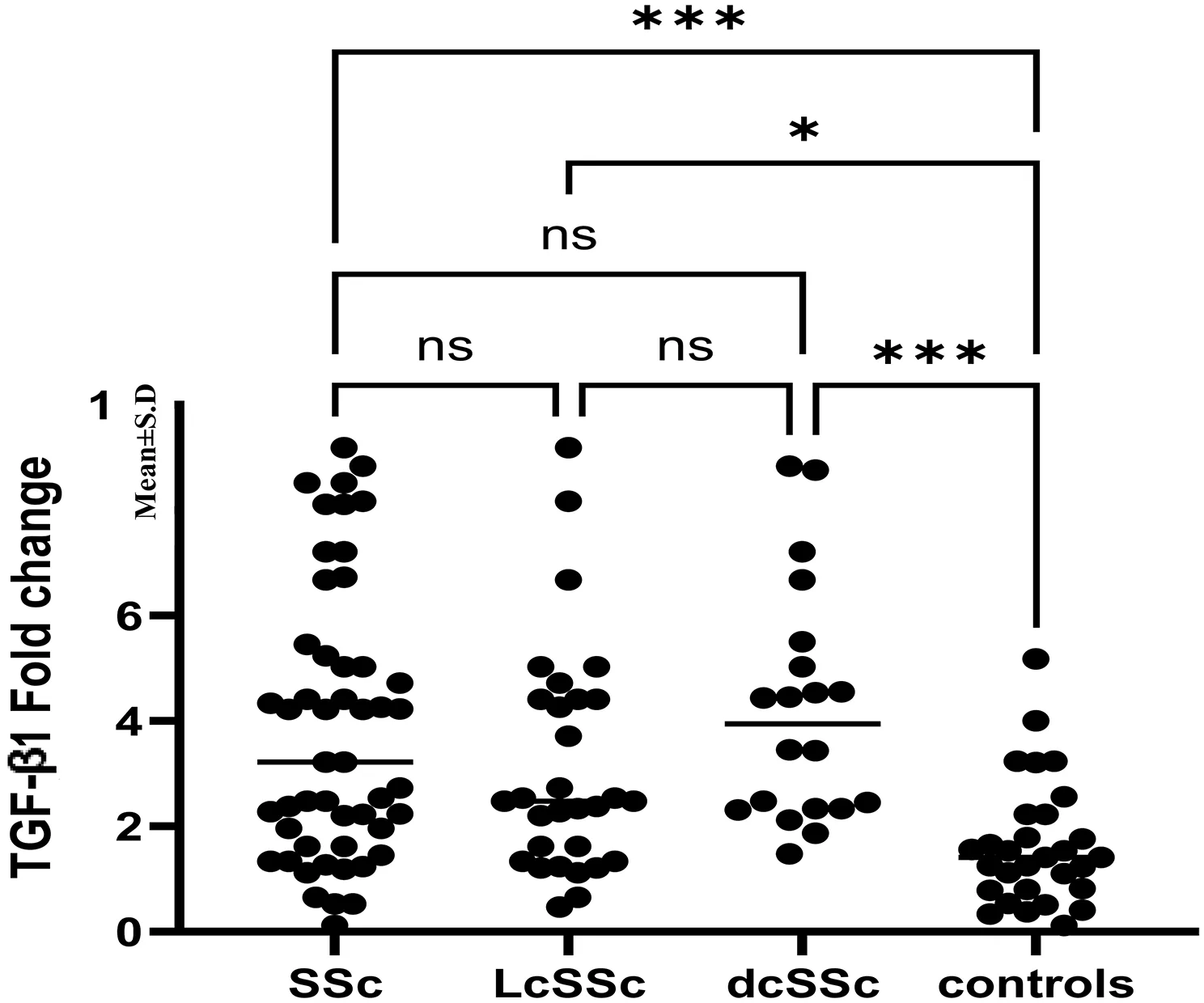
mRNA expression in SSc cases and controls and the SSc subsets in 50 SSc cases & 30 healthy controls. (Analysed by t-test and one-way ANOVA; *P=<0.01; **P=<0.001; ***P=<0.0001, ns: non-significant, lcSSc: limited cutaneous SSc, dcSSc: diffuse cutaneous SSc)

TGF-*β*1 mRNA expression differed significantly between SSc patients (Mean ± S.D. of 3.86 ± 2.56; *N* = 50; Fig. 2) and healthy controls (Mean ± S.D. of 1.64 ± 1.15; *N* = 30; Fig. 2). Cases were further stratified into disease subsets and found that both dcSSc and lcSSc subsets have increased mRNA expression compared to healthy controls. However, the dcSSc group has higher mRNA expression than the lcSSc subset (Fig. 2). The current study found no correlation between the clinical symptoms and mRNA levels.

### Serum levels of active TGF-*β*1

#### Quantitative analysis of transforming growth factor (TGF-*β*1)

**Fig. 3:**
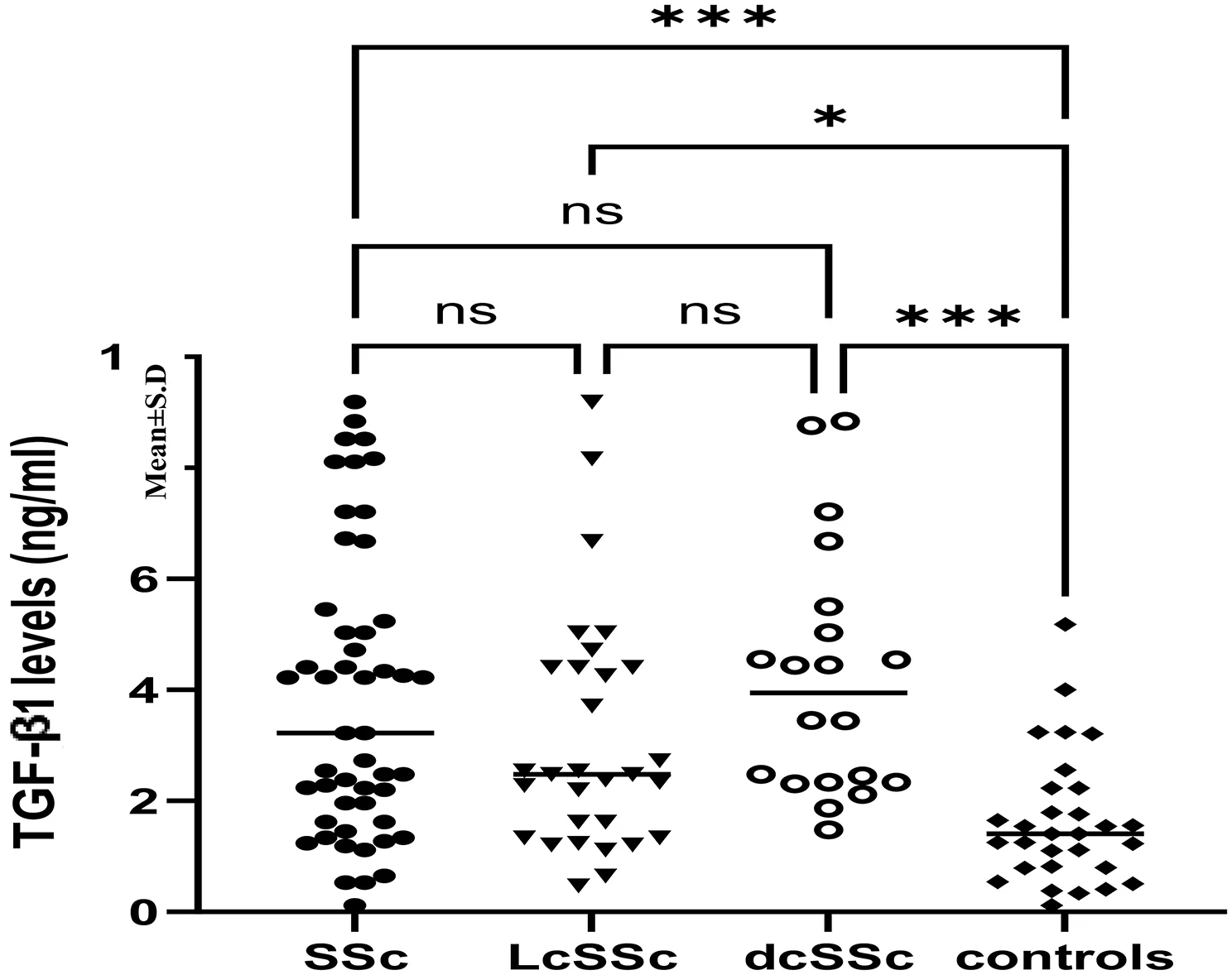
Serum TGF-β1 levels in SSc cases and controls and the SSc subsets in 50 SSc cases & 30 healthy controls. (Analysed by t-test and one-way ANOVA; *P=<0.01; **P=<0.001; ***P=<0.0001, ns: non-significant, SSc: Systemic sclerosis, lcSSc: limited cutaneous Systemic sclerosis, dcSSc: diffuse cutaneous Systemic sclerosis)

The circulating TGF-*β*1 levels in SSc cases were (Mean ± S.D. of 79.21 ± 22.15 ng/ml (*p* < 0.01); *N* = 50; Fig. 3) which was significantly increased in comparison to controls (62.49 ± 15.22ng/ml; *N* = 30; Fig. 3). When SSc patients were classified into lcSSc and dcSSc, both lcSSc patients [Mean ± S.D. of 77.72 ± 22.15; *N* = 12] and dcSSc [Mean ± S.D. of 82.32 ± 21.91; *N* = 38] patients had significantly higher serum TGF-*β*1 levels compared to healthy controls (*p* < 0.05). Patients with a diffuse cutaneous subset of SSc had considerably greater active serum TGF-*β*1 levels than those with the limited subset. Still, no significant difference was found in serum TGF-*β*1 levels between the SSc subsets (Fig. 3).

#### Association of active TGF-*β*1 levels with clinical manifestations

**Fig. 4:**
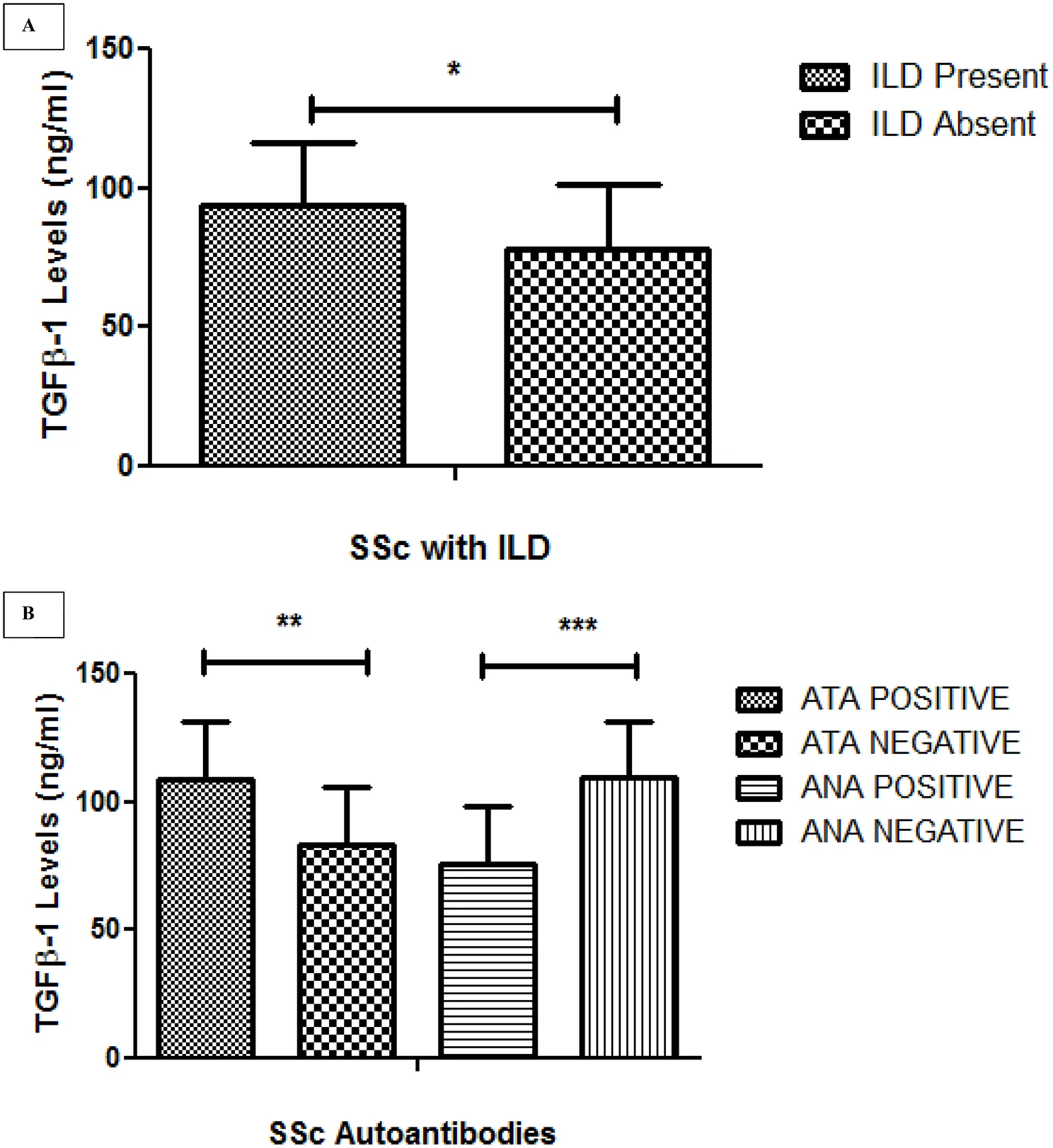
**A** & **B** Association of TGF-β1 with SSc autoantibodies and clinical features in 50 SSc cases & 30 healthy controls. (Analysed by t-test and one-way ANOVA; *P=<0.01; **P=<0.001; ***P=<0.0001; ATA: anti-topoisomerase antibody, ANA: anti-nuclear antibody, ILD: interstitial lung disease, SSc: systemic sclerosis)

To evaluate the correlation between active serum TGF-*β*1 levels and clinical symptoms, patients were categorized according to the presence or absence of the major clinical manifestations. We observed that TGF-*β*1 levels were greater in SSc patients with ILD (*p* < 0.05: Fig. 4A), and digital ulcers. Figure 4 (A & B) represents the association of TGF-*β*1 with SSc autoantibodies and clinical features in 50 SSc cases & 30 healthy controls.

#### Active TGF-*β*1 levels are associated with SSc autoantibodies

Blood TGF-*β*1 levels were greater in SSc patients with positive ATA (*p* < 0.001), and positive ANA antibody (*p* < 0.01). ATA positivity was significantly associated with increased TGF-*β*1 levels in ATA-positive patients (*p* < 0.001; Fig. 4B).

### Correlational analysis

**Fig. 5:**
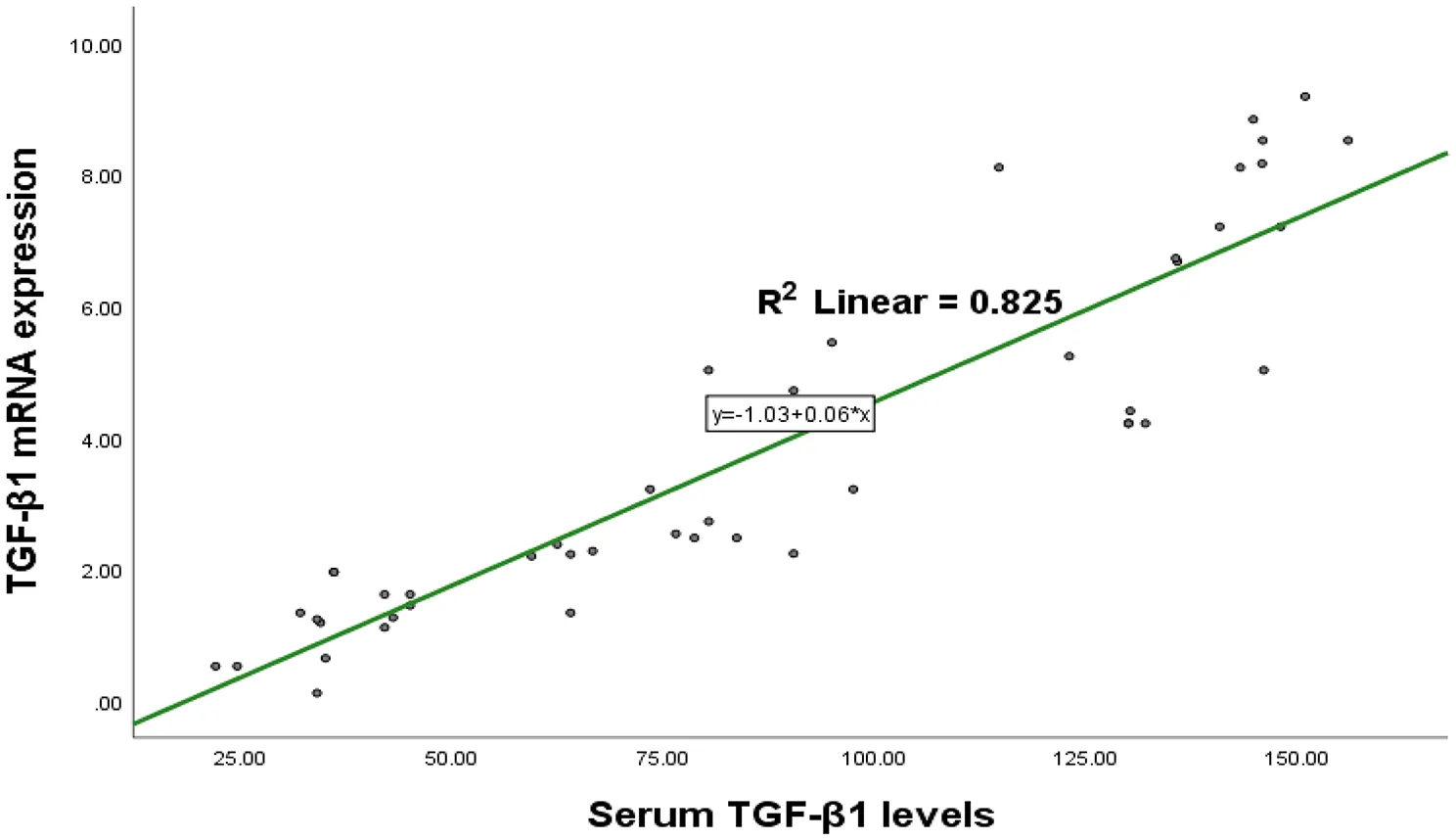
Correlational analysis of TGF-β1 mRNA and serum levels in SSc patients

Bivariate correlation was used to determine whether there is a link between mRNA and serum levels, and it revealed a significant positive correlation between the two variables (*r* = 0.82; *p* < 0.01). Figure 5 depicts the Correlational analysis of TGF-*β*1 mRNA and serum levels in SSc patients.

## Discussion

Cytokine TGF-*β*1 plays a pivotal role in suppressing immune response by promoting the generation of Treg cells. TGF-*β*1 is a significant modulator of fibrosis in SSc [19]. Even though the issue is still controversial in the literature, several research studies have indirectly suggested that TGF-*β*1 levels could be beneficial as a biomarker. This became apparent when they assessed the decrease in TGF-*β*1 following specific treatments (20–21). The effectiveness of serum TGF-*β*1 has been the subject of conflicting findings in earlier investigations [22, 23]. In SSc, TGF-*β*1 has attracted attention as a potential diagnostic marker and as a potential therapeutic target. This study aimed to evaluate TGF-*β*1 mRNA expression and levels in serum and speculate on its potential contribution to SSc risk in the Kashmiri (North-Indian) population. In SSc patients, we found that mRNA expression and serum TGF-*β*1 levels were noticeably greater. Our findings are consistent with those other authors have proposed [24–27]. In addition, the current study also reported a scenario whereby TGF-*β*1 was elevated in subjects with SSc, especially in the dcSSc subgroup, compared to healthy controls. Some investigations, however, did not find any differences between the TGF-*β*1 levels of SSc patients and controls, or even lower categories [28, 29]. Due to variations in the study population (disease duration, therapy), methods employed, and molecules analyzed (total active TGF-*β*1), these studies may have produced different results. Dantas et al. [30] found that patients with dcSSc often had greater serum TGF-*β*1 levels than those with lcSSc. However, we found a significant correlation between elevated serum TGF-*β*1 levels, and the disease subgroup, as well as skin involvement, lung fibrosis, digital ulcers, and positive anti-topoisomerase I antibodies.

TGF-*β*1 is known to play a role in the progression of pulmonary fibrosis, although its function as a biomarker of lung disease is still unknown. In prior investigations, increased TGF-*β*1 serum levels have been found in individuals with idiopathic pulmonary fibrosis [29, 31]. Furthermore, there is conflicting evidence about elevated TGF-*β*1 expression in the lung tissues of patients with SSc. SSc-ILD is a complicated diagnosis, so to provide more specific treatment, new biomarkers must be developed to identify patients early. Here in our study, TGF-*β*1 levels were noticeably greater in patients with ILD compared to healthy controls. We noticed greater TGF-*β*1 levels in individuals with positive anti-topo I, which supports our finding that there is a correlation between TGF-*β*1 levels and the dcSSc subgroup and pulmonary fibrosis and suggests that serum levels might predict the evolution of SSc-ILD. Patients with digital ulcers had greater amounts of TGF-*β*1, according to our research. TGF-*β*1 can also contribute to vascular abnormalities in SSc, even though it has historically been seen as a key fibrosis mediator. **In conclusion**, our analysis suggests that the upregulation of TGF-*β*1 levels in SSc patients plays an important role in the observed vascular and fibrotic abnormalities, and could serve as a marker to identify SSc with chronic fibrotic manifestations.

### Limitation of the Study

A major limitation of the study was the relatively small sample size of the study group in the current study due to the low prevalence of Systemic sclerosis in our population and also due to a lack of resources. Additional research with larger study subjects and diverse ethnicities would be beneficial to ascertain the association between TGF-*β*1 and particular autoantibody responses with clinical symptoms and disease subgroups of Systemic Sclerosis.

## Data Availability

The datasets generated and/or analyzed during the current study are available from the corresponding author on reasonable request.
